# Editorial: AI-assisted bioinformatics and functional genomics technologies in medicinal plants

**DOI:** 10.3389/fpls.2026.1924529

**Published:** 2026-08-10

**Authors:** Cheng Song, Yunpeng Cao, Irfan Ali Sabir, Wanli Zhao

**Affiliations:** 1 College of Life and Health, West Anhui University, Luan, China; 2 College of Forestry, Guangxi University, Nanning, China; 3 College of Horticulture, South China Agricultural University, Guangzhou, China; 4 Institute of Botany, Jiangsu Province and Chinese Academy of Sciences, Nanjing, China

**Keywords:** artificial intelligence, bioinformatics, functional genomic, medicinal plant, regulatory framework

## 1 Introduction

The integration of artificial intelligence (AI) with bioinformatics and functional genomics has triggered a significant shift in medicinal plant studies. This Research Topic unites nine innovative studies that exemplify the scope and depth of this technological transformation (Song et al., 2025). The contributions cover a diverse array of activities, including the development of essential genomics resources, the functional characterization of important gene families, predictive ecological modelling, and the clarification of intricate regulatory frameworks. It is evident that AI and bioinformatics are not just accelerating research. They are also fundamentally altering our comprehension of medicinal plant biology. This evolution has opened up possibilities for precision breeding, increased metabolite production, and sustainable use of these invaluable genetic resources. The arrangement of these contributions into three hierarchical tiers follows a thematic approach, progressing from foundational resources to comprehensive systems-level functional investigations (Borgohain et al., 2026).

## 2 Foundational genomics resources and ecological prediction

The foundation of the research pyramid offers essential genomics blueprints and predictive ecological frameworks. Sudarmono et al. provide a crucial genomics resource in their report, which presents the complete chloroplast genome assembly and annotation of *Helicteres isora* L., a medicinal plant of considerable ethnobotanical significance. Chloroplast genomes, characterized by their conserved structure and uniparental inheritance, are vital tools for phylogenetic reconstruction, species identification, and evolutionary studies. This high-quality assembly enriches the genomic database for Helicteres and provides a robust molecular marker resource for conservation genetics and authentication of this medicinally valuable species—a critical step given the challenges of adulteration in herbal supply chains. Wang et al. used maximum entropy (MaxEnt) modeling to predict potential suitable habitats for three species of *Trichosanthes* L. in China under future climate change scenarios, thus moving from static genomic resources to dynamic ecological predictions. *Trichosanthes* species are economically important medicinal plants and their future geographic distribution is important for conservation planning and sustainable cultivation. Using the MaxEnt model, a machine learning technique, we predicted changes in habitat suitability under different climate scenarios by combining occurrence data and bioclimatic variables. It was demonstrated that how AI-assisted ecological niche modeling can be used to inform adaptive management strategies for ensuring the long-term availability of critical medicinal plant resources under rapid environmental change.

## 3 Genome-wide identification and characterization of functional gene families

The second tier encompasses studies that systematically identify and characterize large gene families central to stress responses and metabolic regulation (Labbé et al., 2024). These genome-wide analyses, facilitated by advanced bioinformatics pipelines, provide the essential foundation for functional genomics. Chen et al. conducted a comprehensive investigation into the *SOS1* gene family in *Paeonia ostii*, encompassing its genome-wide identification, characterization, and expression analysis under salt stress conditions. *SOS1* genes are responsible for encoding plasma membrane Na^+^/H^+^ antiporters, which are vital for salt tolerance. This trait is of increasing importance due to the threat of soil salinization to the cultivation of medicinal plants. The key *SOS1* members involved in salt stress were identified using phylogenetics, motif analysis, and expression profiling under salt treatment. The study provides valuable candidate genes for improving salt tolerance in *P. ostii* through genetic engineering or marker-assisted breeding. Fan et al. systematically identified and analyzed WRKY transcription factors in *Myrica rubra*, a fruit tree with medicinal properties. WRKY proteins form one of the largest plant transcription factor families and are master regulators of stress responses, development, and secondary metabolism (Zhang et al., 2023). The authors identified 55 *WRKY* genes, elucidated their phylogenetic relationships, gene structures, and conserved motifs, and importantly, demonstrated the positive regulatory role of *MrWRKY14* in sugar synthesis. MrWRKY14 was shown to significantly activate the promoter of the *SWEET1* gene, a sugar transporter, suggesting its involvement in sugar accumulation during fruit development. This discovery not only advances our understanding of sugar-acid regulatory networks but also has implications for improving fruit quality and, by extension, the medicinal value of *M. rubra*. Zhang et al. further extended this gene family analysis to the NAC transcription factors in *Eriobotrya japonica*, focusing on their implications for sugar-acid regulatory networks during fruit development. NAC proteins are plant-specific transcription factors involved in numerous developmental processes and stress responses. Through the integration of genome-wide identification with expression analysis during fruit maturation, the authors have identified potential NAC members that may orchestrate the balance between sugar accumulation and acid degradation. This balance is a key determinant of fruit flavor and nutritional quality. The present study provides a foundation for manipulating fruit quality traits in loquat and other fruit-bearing medicinal plants. Xiang et al.contributed a genome-wide analysis and functional characterization of terpene synthase (TPS) genes in *Angelica sinensis*, which has been used for centuries in traditional Chinese medicine. *TPS* genes are pivotal enzymes in terpenoid biosynthesis, responsible for the substantial chemical diversity of this class of natural products, including numerous pharmacologically active compounds. Through genome-wide mining, phylogenetic classification, and expression analysis, the authors identified *AsTPS* genes potentially involved in the biosynthesis of bioactive terpenoids. This study provides a comprehensive list of candidate genes for metabolic engineering, with the aim of enhancing the production of therapeutic terpenoids in *A. sinensis*.

## 4 Integrative functional genomics and systems-level regulation

The third and most integrative tier features studies that combine transcriptomics, metabolomics, and functional assays to dissect complex regulatory landscapes and metabolic pathways. These contributions exemplify the power of multi-omics integration in revealing the intricate mechanisms underlying stress adaptation and secondary metabolite production. Zhang et al. presented a multi-layer regulatory landscape of chilling requirement in peach floral buds, employing stage-resolved transcriptomics and hormone profiling. Chilling is a critical trait for dormancy release and flowering in temperate fruit trees, and its understanding is essential for adapting cultivation to changing climates. While peach is primarily a fruit crop, its buds have been used in traditional medicine, and the underlying regulatory mechanisms may inform studies in other medicinal woody plants. By integrating time-series transcriptomics with hormone measurements and employing network inference algorithms, the authors constructed gene co-expression networks and identified key transcription factors and hormone signaling modules orchestrating chilling responses. This multi-omics approach provides a systems-level view of a complex adaptive trait, offering a methodological blueprint for studying similar processes in medicinal plants. Wang et al.conducted a functional genomics investigation of CYP71AJ49 in *Peucedanum praeruptorum*, a medicinal plant known for its coumarin-rich roots. Through *Agrobacterium*-mediated overexpression, the authors revealed the dual roles of this cytochrome P450 enzyme in drought adaptation and coumarin biosynthesis. This elegant study demonstrates how functional genomics can unravel the pleiotropic functions of a single gene, linking stress responses to secondary metabolism. The dual functionality of CYP71AJ49 suggests a metabolic crosstalk between drought stress signaling and coumarin production, with implications for both stress tolerance and metabolite yield. The findings highlight the potential of engineering such pleiotropic genes to simultaneously improve stress resilience and the production of bioactive compounds. Moreover, Song et al. provides a comprehensive overview of the AI-assisted technologies driving this transformation. The authors articulate how AI is revolutionizing genome annotation, predicting protein structures, reconstructing metabolic pathways, and integrating multi-omics data. Deep learning is highlighted as a game-changer, enabling the analysis of high-throughput data with unprecedented accuracy and speed. For example, the application of deep learning in single-cell genomics and gene regulatory network inference opens new ways for understanding cell-type-specific gene expression and the complex regulatory cascades governing metabolite production (Xu et al., 2025).

Collectively, these studies provide a comprehensive overview of the current state and future trajectory of medicinal plant research. Firstly, there is the integration of artificial intelligence and machine learning into genomics workflows. Secondly, there is the integration of multi-omics datasets to capture biological complexity. Thirdly, fundamental research is to be integrated with applied objectives such as breeding, conservation, and sustainable production. However, the integration of AI with bioinformatics and functional genomics signifies more than a mere incremental advancement, which represents a paradigm shift in our ability to decipher the genetic and molecular underpinnings that govern these invaluable plant resources. The studies are ranging from foundational genome assemblies to integrative functional analyses, collectively underscore the power of this interdisciplinary approach. As the refinement of AI algorithms, expansion of genomics database, and development of more sophisticated integration frameworks continue, the full therapeutic potential of medicinal plants is on the verge of being unlocked. This may result in a transformation of the way in which natural products is discovered, cultivated, and utilized for the benefit of global healthcare and sustainable development.
